# Metabolite interactions mediate beneficial alliances between *Bacillus* and *Trichoderma* for effective *Fusarium* wilt control

**DOI:** 10.1093/ismejo/wraf283

**Published:** 2025-12-27

**Authors:** Jiyu Xie, Xinli Sun, Tao Wen, Yaoqiang Bai, Tong Qian, Shunjuan Hu, Lihao Chen, Pan Wang, Youzhi Miao, Ruifu Zhang, Ákos T Kovács, Zhihui Xu, Qirong Shen

**Affiliations:** Jiangsu provincial key lab for solid organic waste utilization, Key lab of organic-based fertilizers of China, Jiangsu Collaborative Innovation Center for Solid Organic Wastes, Educational Ministry Engineering Center of Resource-saving fertilizers, Nanjing Agricultural University, Nanjing, Jiangsu 211800, China; Institute of Biology Leiden, Leiden University, Leiden, 2333 BE, The Netherlands; Jiangsu provincial key lab for solid organic waste utilization, Key lab of organic-based fertilizers of China, Jiangsu Collaborative Innovation Center for Solid Organic Wastes, Educational Ministry Engineering Center of Resource-saving fertilizers, Nanjing Agricultural University, Nanjing, Jiangsu 211800, China; Jiangsu provincial key lab for solid organic waste utilization, Key lab of organic-based fertilizers of China, Jiangsu Collaborative Innovation Center for Solid Organic Wastes, Educational Ministry Engineering Center of Resource-saving fertilizers, Nanjing Agricultural University, Nanjing, Jiangsu 211800, China; Jiangsu provincial key lab for solid organic waste utilization, Key lab of organic-based fertilizers of China, Jiangsu Collaborative Innovation Center for Solid Organic Wastes, Educational Ministry Engineering Center of Resource-saving fertilizers, Nanjing Agricultural University, Nanjing, Jiangsu 211800, China; Jiangsu provincial key lab for solid organic waste utilization, Key lab of organic-based fertilizers of China, Jiangsu Collaborative Innovation Center for Solid Organic Wastes, Educational Ministry Engineering Center of Resource-saving fertilizers, Nanjing Agricultural University, Nanjing, Jiangsu 211800, China; Jiangsu provincial key lab for solid organic waste utilization, Key lab of organic-based fertilizers of China, Jiangsu Collaborative Innovation Center for Solid Organic Wastes, Educational Ministry Engineering Center of Resource-saving fertilizers, Nanjing Agricultural University, Nanjing, Jiangsu 211800, China; Jiangsu provincial key lab for solid organic waste utilization, Key lab of organic-based fertilizers of China, Jiangsu Collaborative Innovation Center for Solid Organic Wastes, Educational Ministry Engineering Center of Resource-saving fertilizers, Nanjing Agricultural University, Nanjing, Jiangsu 211800, China; Jiangsu provincial key lab for solid organic waste utilization, Key lab of organic-based fertilizers of China, Jiangsu Collaborative Innovation Center for Solid Organic Wastes, Educational Ministry Engineering Center of Resource-saving fertilizers, Nanjing Agricultural University, Nanjing, Jiangsu 211800, China; Jiangsu provincial key lab for solid organic waste utilization, Key lab of organic-based fertilizers of China, Jiangsu Collaborative Innovation Center for Solid Organic Wastes, Educational Ministry Engineering Center of Resource-saving fertilizers, Nanjing Agricultural University, Nanjing, Jiangsu 211800, China; Jiangsu provincial key lab for solid organic waste utilization, Key lab of organic-based fertilizers of China, Jiangsu Collaborative Innovation Center for Solid Organic Wastes, Educational Ministry Engineering Center of Resource-saving fertilizers, Nanjing Agricultural University, Nanjing, Jiangsu 211800, China; Institute of Biology Leiden, Leiden University, Leiden, 2333 BE, The Netherlands; Jiangsu provincial key lab for solid organic waste utilization, Key lab of organic-based fertilizers of China, Jiangsu Collaborative Innovation Center for Solid Organic Wastes, Educational Ministry Engineering Center of Resource-saving fertilizers, Nanjing Agricultural University, Nanjing, Jiangsu 211800, China; Jiangsu provincial key lab for solid organic waste utilization, Key lab of organic-based fertilizers of China, Jiangsu Collaborative Innovation Center for Solid Organic Wastes, Educational Ministry Engineering Center of Resource-saving fertilizers, Nanjing Agricultural University, Nanjing, Jiangsu 211800, China

**Keywords:** *Bacillus*, *Trichoderma*, surfactin, fusaric acid, BFIs, *Fusarium*

## Abstract

Bacteria-Fungi Interactions play a crucial role in soil nutrient cycling and plant disease suppression. *Bacillus* and *Trichoderma* exhibit antagonism when inoculated on laboratory media, global soil sample analysis reveals a positive correlation between these two genera in addition to enhanced plant-pathogen *Fusarium oxysporum* suppression and plant growth promotion. Here, we assess cross-kingdom interactions within artificial model communities of *Bacillus velezensis* and *Trichoderma guizhouense*. Transcriptomic profiling revealed that in the presence of fungi, the key stress sigma factor of *B. velezensis* activates expression of biosynthetic genes for antimicrobial secondary metabolite production. Among these, surfactin induces T22azaphilone production in *T. guizhouense* that hinders oxidative stress. Both surfactin and T22azaphilone contribute to *Bacillus* and *Trichoderma* maintenance in soil in the presence of *F. oxysporum*. Finally, *F. oxysporum*-secreted fusaric acid temporarily inhibits *B. velezensis* growth whereas it is efficiently degraded by *T. guizhouense*. These metabolite-mediated interactions reveal how competing soil microorganisms could form effective alliances that ultimately enhance plant protection against soil-borne pathogens.

## Graphical Abstract

**Graphical Abstract:**
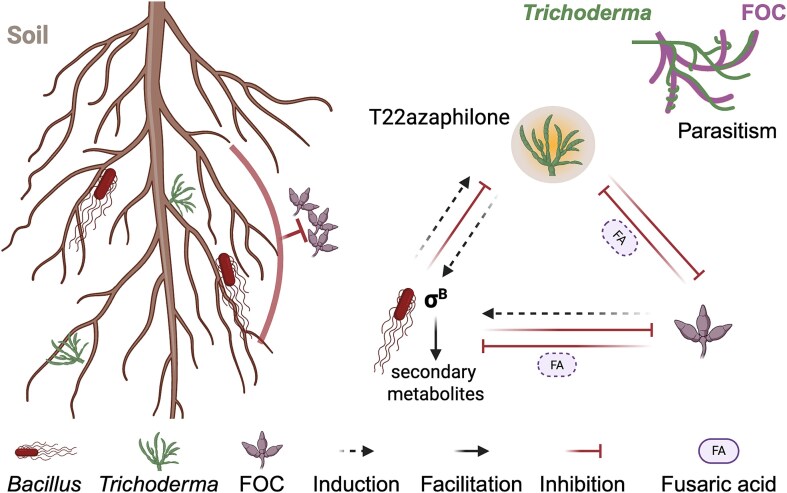

## Introduction

Chemical signaling orchestrates the complex interactions between beneficial bacteria, fungi, and plant pathogens within the soil microbiome [1, 2]. Understanding how potentially competing species achieve stable community assembly in these environments is essential for developing effective biological control strategies. Species may co-occur through resource partitioning, mutual facilitation, or cooperative responses to shared threats that reduce direct competitive pressure [3, 4].

*Fusarium oxysporum* represents a shared threats to beneficial soil microorganisms in agricultural systems, causing devastating losses in economic crops such as cucumber, banana, watermelon, and others [5]. This soil-borne pathogen is equipped with multiple mechanisms for pathogenesis and persistence: it produces phytotoxins such as fusaric acid which contribute to plant wilting and root damage [6], forms resistant chlamydospores that enable long-term soil survival under adverse conditions [7], and colonizes the plant vascular system to disrupt water and nutrient transport. Traditional chemical control methods have proven increasingly insufficient due to rapid resistance development, limited soil penetration, and growing environmental and health concern [8]. These limitations have created urgent need for sustainable biological control strategies.

Cross-kingdom interactions between bacteria and fungi are key drivers of soil health. Through complex metabolic and physical exchanges, they shape microbial communities and influence critical ecosystem functions [9–12]. These complex interactions encompass both direct physical contact, such as bacterial colonization on fungal hyphae and biofilm formation [13, 14], indirect chemical communication through secondary metabolites [15], and resource competition that can either promote or inhibit community members [16]. Among promising biocontrol agents, the bacterium *Bacillus velezensis* suppresses soil-borne pathogens through production of antimicrobial lipopeptides including surfactin, fengycin, bacillomycin D, whereas simultaneously promoting plant growth through phosphate solubilization, siderophore production, and recruitment of other beneficial microorganisms [17, 18]. Similarly, the fungus *Trichoderma guizhouense* combats pathogenic fungi through mycoparasitism, competitive nutrient acquisition, and hydrolytic enzyme production [15, 16], particularly chitinase, whereas also enhancing plant development through bioactive compound production and root colonization [19–21]. Despite documented individual efficacy, current understanding remains limited by focus on pairwise interactions rather than multispecies ecological networks that better represent natural soil conditions.

Current understanding of beneficial microbial interactions is complicated by apparent contradictions between laboratory and field observations. Whereas *B. velezensis* and *T. guizhouense* exhibit mutual antagonism in laboratory conditions [14], agricultural applications demonstrate that *Bacillus* and *Trichoderma* species can successfully co-occur for enhanced biocontrol efficacy [22–24]. This discrepancy suggest that natural soil environments provide conditions that enable competing species to co-occur despite direct antagonistic potential. In complex soil environments, competing species may achieve co-occurrence through resource partitioning, mutual facilitation, or indirect cooperation mediated by shared enemies. Understanding the molecular basis of such ecological flexibility is crucial for developing effective biocontrol strategies.

Here, we investigated whether secondary metabolite-mediated mechanisms could explain the ecological co-occurrence of plant-beneficial microorganisms by examining their interactions in a tri-species system with *F. oxysporum* f. sp. *cucumerinum* (FOC) as a shared target. We hypothesized that pathogen presence would alter the competitive interaction between *B. velezensis* SQR9 and *T. guizhouense* NJAU 4742, potentially enabling stable co-occurrence through coordinated stress responses and metabolite-mediated communication. Using integrated transcriptomic profiling, targeted mutant analysis, and liquid chromatography-mass spectrometry (LC–MS), we demonstrate that bacterial stress regulation via σ^B^ (sigma B, a secondary sigma factor of *Bacilli*) coordinates secondary metabolite production in *Bacillus*, surfactin mediates cross-kingdom facilitation by inducing protective compound synthesis in *Trichoderma*, and pathogen-derived fusaric acid creates temporal dynamics that support co-occurrence of *Bacillus* and *Trichoderma*. These findings reveal how molecular mechanisms underlie ecological principles governing microbial community interaction and provide foundations for rational biocontrol consortium design.

## Materials and methods

### Metagenomic analysis

Soil metagenomic datasets (n = 1680) were retrieved from NCBI SRA database through systematic literature screening. Dataset selection criteria: (i) metagenomic sequencing from soil environments, (ii) sufficient sequencing depth, and (iii) available metadata including geographic location and soil type. Studies involving extreme environments and laboratory-treated samples were excluded. Complete sample information was listed in Data S1. The data underwent quality control with FastQC (v.0.11.9) and Trimmomatic v.0.39 (parameters set as: leading: 3, trailing: 3, sliding window: 4:15, minlen: 36), removing bases with quality scores <15 and sequences <36 bp. Cleaned sequences were assembled using MEGAHIT, and employed PROkaryotic DynamIc programming Genefinding ALgorithm (Prodigal) to identify open reading frames (ORFs), dereps generated nonredundant amino acid sequences, and Bowtie2 determined gene abundance. Sequences were annotated using KEGG, Kraken2, and GO databases.

### Strains, growth conditions, and interaction on agar medium

The strains in this study are listed in Table 1. *B. velezensis* SQR9 (Bv, China General Microbiology Culture Collection Center, CGMCC No. 5808, NCBI accession No. CP006890.1) was grown at 30°C in lysogeny broth (LB, Lennox, Carl Roth, Germany) for overnight culture, supplemented with 1.5% Bacto agar if required. *T. guizhouense* NJAU 4742 (Tg, CGMCC No. 12166, NCBI accession No. LVVK01000021.1) and *F. oxysporum f.* sp. *cucumerinum* (FOC, Agricultural Culture Collection of China, ACCC No. 30220) were grown at 28°C in Potato Dextrose Agar (PDA, BD Difco, US) medium for 7 days. Spores were harvested with 5 mL water, subsequently filtered through Miracloth, and stored at 4°*C. Media* were supplemented with selective antibiotics: chloramphenicol (Cm, 5 μg mL^−1^), zeocin (Zeo, 20 μg mL^−1^), spectinomycin (Spec, 100 μg mL^−1^), and hygromycin B (Hyg, 100 μg mL^−1^).

**Table 1:** Bacterial and fungal strains used in this study.

Strains	Genotype	Reference
*B. velezensis* SQR9	Wild-type isolate bacterium	[14]
*B. velezensis* gfp	wild type with pNW33n-gfp, Cm^R^	[14]
*B. velezensis* Δ*sigB*	Δ*sigB*::Spec^R^	This study
*B. velezensis OEsigB*	pNW33N p43-*sigB*, Cm^R^	This study
*B. velezensis* Δ*srf*	Δ*srf*::Zeo^R^	[25]
*B. velezensis* Δ*bac*	Δ*bac*::Zeo^R^	[25]
*B. velezensis* Δ*dfn*	Δ*dfn*::Zeo^R^	[25]
*B. velezensis* Δ*fenA*	Δ*fenA*::Zeo^R^	[25]
T*. guizhouense* NJAU 4742	Wild-type isolate fungus	[14]
*T. guizhouense* mCherry	mCherry, Hyg^R^	[14]
*T. guizhouense* Δ*tga5*	Δ*tga5*::Hyg^R^	[26]
*F. oxysporum f.sp cucumerinum*	Wild type	[27]

Cm^R^, Zeo^R^, Spec^R^, and Hyg^R^ denote chloramphenicol, zeocin, spectinomycin, and hygromycin B resistance markers.

Confrontation assays were performed on PDA medium. To accommodate differential growth rates, 2 μL fungal spore suspension was inoculated at the center of the plate, followed by 24-h incubation at 28°C. Subsequently, 2 μL *B. velezensis* overnight culture diluted to an optical density at 600 nm (OD_600_) of 1 was introduced. Plates were incubated at 28°C for 3-7 days. Media were supplemented with antibiotics or fusaric acid when applicable.

### Plant experiments design

Greenhouse trials were conducted at Nanjing Agricultural University. Soil was collected from a historically cucumber-cultivated field in Nanjing, Jiangsu Province, China, with the following properties: pH 6.2, organic matter 26.1 g kg^−1^, available N 171.3 mg kg^−1^, available P 131.4 mg kg^−1^, available K 238.8 mg kg^−1^, total N 1.9 g kg^−1^, total P 1.7 g kg^−1^ and total K 14.2 g kg^−1^. Cucumber (*Cucumis sativus L. cv.* Jinchun No.4) seeds were surface-disinfected in 2% sodium hypochlorite (3 min), rinsed three times with sterilized distilled water, and germinated on sterile moistened filter paper in 9 cm Petri dishes at 30°C. Following one week of germination, seedlings were transplanted into pots containing 2 kg soil, and cultivated for an additional week. After the one-week establishment, FOC was inoculated first, followed by *B. velezensis* and *T. guizhouense* after seven days.

The experimental design incorporated five treatments: CTL (control, no inoculation); FOC alone; FOC + Bv; FOC + Tg; FOC + Bv + Tg. Spores and bacterial cells were centrifuged (5000 rpm, 5 min), washed twice, and resuspended in sterile deionized water. Spore concentrations were determined using a hemocytometer, whereas bacterial densities were adjusted by OD_600_. Inoculation densities were standardized at 10^7^ spores g^−1^ soil for fungi and 10^8^ CFU g^−1^ soil for bacteria. Plants were maintained under controlled conditions (30/22°C, 16/8 h light/dark cycles) for 8 weeks. Plant height, fresh weight, and dry weight were recorded, with six replicates per treatment. Microbial cell numbers were quantified by qPCR as described below, with six replicates per treatment. Disease index (DI) was assessed using a 6-point scale (0–5) based on leaf yellowing and vascular discoloration: DI (%) = [∑(N × score)]/(5 × T) × 100, where N represents the number of plants at each symptom level, score is the disease grade, and T is the total number of evaluated plants [28].

### Inhibition analysis of microbial interactions

Inhibitory effects of bacterial metabolites on fungal growth were assessed using a 48-well plate assay system [29]. Fungal spores (*T. guizhouense* and FOC) were harvested from 7-day-old PDA cultures, filtered through Miracloth, and adjusted to 10^7^ spores mL^−1^ using a hemocytometer. Cell-free supernatants were obtained from 48-h *B. velezensis* LB cultures by centrifugation (10 000 rpm, 10 min) and sterile filtration (0.22 μm). Each well contained 200 μL Potato Dextrose Broth (PDB, BD Difco, US) medium inoculated with fungal spores (10^6^ spores mL^−1^) and supplemented with bacterial supernatant at graduated volumes (0, 10, 20, 40, 60, 80 μL), using sterile LB medium as the negative control. The cultures were inoculated at 28°C in darkness for 72 h, after which fungal growth was quantified using spectrophotometric analysis. Inhibition potency was expressed through a scoring system derived from the equation: Inhibition Score = 5 - ∑(relative fungal growth).

### Whole-genome transcriptomic analysis and reverse transcription qPCR validation

RNA was extracted from microbial interaction zones (white rectangular boxes in respective figure), scraped from agar surfaces using sterile loops. Samples were processed using Trizol reagent kit (Invitrogen, Carlsbad CA, USA), with RNA quality validated using an Agilent 2100 Bioanalyzer. Eukaryotic mRNA was enriched using Oligo (dT) beads, whereas prokaryotic mRNA was isolated using Ribo-Zero Magnetic Kit (Epicentre). Enriched mRNA was fragmented at 94°C for cDNA library construction. First-strand cDNA was synthesized using reverse transcription, followed by second-strand cDNA synthesis with simultaneous end repair and A-tailing. Adapters were then ligated to the cDNA fragments, followed by size selection using magnetic beads and PCR amplification. Libraries were sequenced on Novaseq 6000 (Illumina) (NCBI SRA database BioProject PRJNA1201065). Following quality trimming, reads were aligned to reference genomes using Bowtie 2-2.2.3 [30]. Differential gene expressions (DGEs) analysis was conducted using DESeq2 [31], with p-values adjusted for false discovery rate (FDR) using the Benjamini-Hochberg procedure. Differential expression criteria were established as log2 fold change (LFC) > 2 and FDR < 0.05. Functional annotation was performed by mapping sequences to KEGG Orthology terms via EggNOG-mapper v2 [32], with Benjamini-Hochberg correction.

RNA samples were reverse-transcribed using PrimeScript RT reagent kit with gDNA eraser (Toyobo). Target genes (*recA, srfAC, bmyA, fenA, baeC, mlnH, dfnX, bacA, dhbF, tef, tga5*, primers in Table S1) were quantified using ChamQ SYBR qPCR Master Mix (Vazyme) on an Applied Biosystems Real-Time PCR system. The *recA* and *tef* served as internal controls for *B. velezensis* and *T. guizhouense*, respectively. The reaction mixture (20 μL) contained: 7.2 μL H₂O, 10 μL 2× ChamQ SYBR qPCR Master Mix (Vazyme), 0.4 μL of each primer (10 μmol L^−1^), and 2 μL template. Thermal cycling conditions comprised initial denaturation at 95°C for 10 min, followed by 40 cycles of 95°C for 30 s and 60°C for 45 s, with a subsequent melting curve analysis. Relative gene expression was calculated using the 2^−ΔΔCT^ method [33].

### Cell number quantification in soil

Sterilized soil (𝛾-irradiation,>50kGray, Xiyue Radiation Technology, Nanjing, China) was distributed into sterile containers (200 g) and maintained without plants under greenhouse conditions. Gamma irradiation was used as it minimizes physicochemical alterations compared to autoclaving [34–36]. Sterilization was verified by culturing soil samples on LB agar and PDA medium; no colonies were detected after 5 days at 28°C. Samples were maintained in sealed containers to prevent contamination. The soil used was identical to plant experiments to ensure consistency. Soil was inoculated with *B. velezensis* (10^8^ CFU g^−1^ soil) and *T. guizhouense*/FOC (10^7^ spores g^-1^ soil) as described above. Samples were collected at 4-day intervals for DNA extraction using DNeasy Power Soil Pro Kit (Qiagen). Cell numbers were quantified by qPCR [28, 37–39] using primers targeting single-copy genes identified through comparative genomic analysis using Roary (primers in Table S1). Primer specificity was validated by melting curve analysis (single peaks) and standard curve performance (*R*^2^ > 0.99, efficiency 90%–110%) (Fig S1). The amplified fragments were cloned into pMD19T vectors to generate standard curves. qPCR was performed as described above. Each treatment had six replicates. All samples were processed identically using the same soil batch, ensuring consistent PCR conditions across treatments. Data represent relative abundances suitable for comparing population dynamics among treatments.

### Construction of mutant and overexpression strains

The *sigB* deletion mutant was generated using an overlap PCR-based strategy [27]. The upstream and downstream regions of *sigB* were amplified from wild-type genomic DNA by primer pairs sigB_UF/UR and sigB_DF/DR, respectively (Table S1). The spectinomycin resistance cassette was amplified from plasmid pheS-SPC using primers Spc_F/R. These three fragments were joined using overlap PCR (upstream-antibiotic marker-downstream). For overexpression strain construction, *sigB* gene was amplified using primers OEsigB_F/R and cloned into plasmid pNW33N containing the p43 promoter via EcoRI and BamHI restriction sites. The constructs were directly transformed into *B. velezensis,* selected on LB agar medium with appropriate antibiotics*.* All constructed strains were verified by DNA sequencing.

### Growth curve analysis

The growth dynamics of *B. velezensis* were monitored in 96-well plates (VWR), with each well containing 200 μL LB medium inoculated with 2 μL overnight culture (OD_600_ = 1.0). Growth measurements (OD_600_) were recorded at 10-min intervals for 24/48 h at 30°C using an Agilent Synergy H1 microplate reader (Biotek).

For coculture experiments, *B. velezensis* and *T. guizhouens*e were cultivated in 24-well plates (VWR) with 2 mL LB medium per well. Each well was inoculated with 1% (v/v) *B. velezensis* (OD_600_ = 1.0) and *T. guizhouense* (10^8^ spores mL^−1^). Growth parameters were monitored over 72 h at 30°C using scanning mode measurements of OD_600_, GFP fluorescence (excitation/emission: 485/528 nm), and mCherry fluorescence (excitation/emission: 590/635 nm) at 10-min intervals. Different concentration of fusaric acid was added if required. Each treatment had four replicates.

### T22azaphilone quantification

T22azaphilone was quantified by solvent extraction followed by high-performance liquid chromatography (HPLC). Culture plates from interaction assays were transferred to flasks and mechanically disrupted. Metabolites were extracted twice with ethyl acetate (TEDIA, USA) at a 1:2 volume ratio. Organic phases were combined, concentrated under N_2_, and reconstituted in methanol. HPLC was conducted using a Waters e2695 equipped with an XBridge C18 column (5 μm, 4.6 × 250 mm) at 30°C with a flow rate of 1 mL min^−1^, employing a linear gradient of acetonitrile/water (containing 0.1% formic acid) from 5% to 95%. T22azaphilone was identified at 22.5 min and quantified by peak area analysis, each treatment had five replicates.

### Fusaric acid degradation

Commercial fusaric acid (Sigma–Aldrich 55 952) was validated against FOC-produced fusaric acid by HPLC (Fig S2). *T. guizhouense* spores (10^7^ spores mL^−1^, 1% v/v) were cultivated in 100 mL minimal medium (MM medium, 10 g L^−1^ glucose, 5 g L^−1^ (NH_4_)_2_SO_4_, 0.6 g L^−1^ MgSO_4_·7H_2_O, 15 g L^−1^ KH_2_PO_4_, 0.05 g L^−1^ CaCl_2_, 0.5 mL L^−1^ trace element stock) at 180 rpm, 28°C for 72 h. FeSO_4_·7H_2_O (0.5 g), MnSO_4_·2H_2_O (0.16 g), ZnSO_4_·7H_2_O (0.14 g), and CoCl_2_·6H_2_O (0.2 g) were dissolved in 50 mL distilled water for the trace element stock. Metabolite extraction was performed by combining 1 mL culture supernatant with 1 mL isopropanol: ethyl-acetate (1:3 v/v) containing 1% formic acid, desiccated under N_2_, and reconstituted in methanol. Chromatographic separation used an Agilent 1290 UHPLC system coupled to an Agilent 6545 QTOF MS with Dual Jet Stream ESI. Samples (1 μL) were separated on a Poroshell 10 Phenyl Hexyl column (250 × 2.1 mm, 2.7 μm; Agilent Technologies) maintained at 40°C using acetonitrile/water (buffered with 20 mmol L^−1^ formic acid) at 0.35 mL min^−1^, with a linear gradient 10-100% acetonitrile over 15 min, followed by a 2-min hold, and 0.1-min re-equilibration. Data were analyzed using Agilent MassHunter Qualitative Analysis software.

### NBT and DAB staining methods

For superoxide (O^2−^) detection, Nitro Blue Tetrazolium (NBT, Aladdin, Shanghai) staining solution was prepared (0.05% NBT in 50 mM phosphate buffer, pH 7.5). For peroxide detection, Diaminobenzidine (DAB, Aladdin, Shanghai) staining solution was prepared (2.5 mM DAB with horseradish peroxidase (Aladdin, Shanghai)) at 5 purpurogallin units mL^−1^ in 50 mmol L^−1^ phosphate buffer, pH 6.5). 10 mL staining solution was applied to each plate and incubated with agitation (100 rpm) at room temperature for 30 min. After removing staining solutions, plates were maintained at 25°C in darkness for 7 h before imaging. The intensity of blue precipitate in NBT-stained samples indicated O^2−^ levels, whereas brown precipitate in DAB-stained samples reflected the peroxide levels.

**Figure 1:**
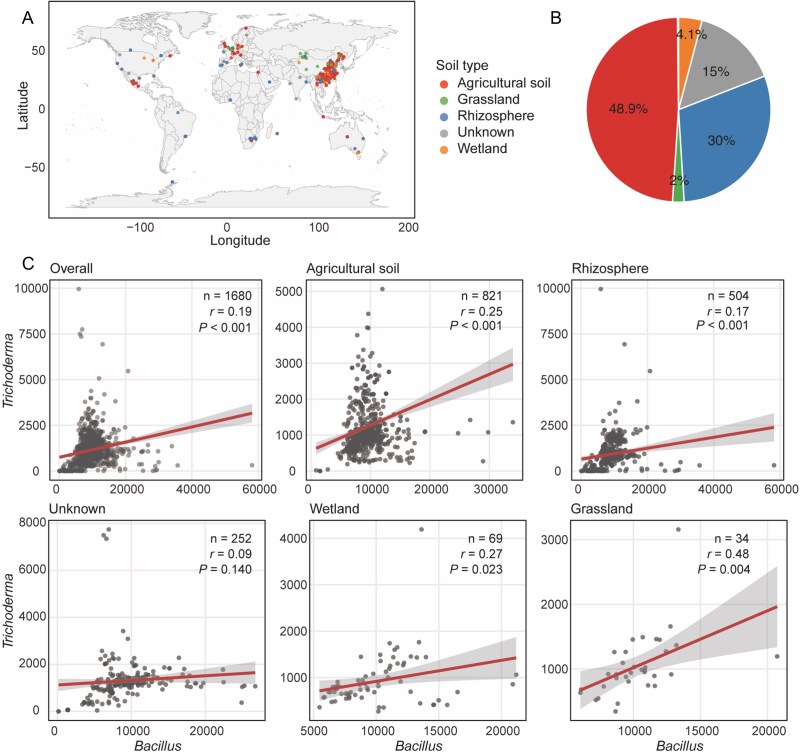
Positive correlation between *Bacillus* and *Trichoderma* genera across global soil metagenomes. (**A**) Geographic distribution of 1680 soil metagenome samples colored by soil type. (**B**) Proportion of samples from different soil environments (n = 1680). (**C**) Pearson correlation analysis between *Bacillus* and *Trichoderma* relative abundances in overall dataset and stratified by soil environment. Each point represents one soil sample. Red lines show linear regression with 95% confidence intervals (gray shading). Sample size (n), correlation coefficient (*r*), and *P* values are indicated.

### Statistical analysis

Analysis and figure preparation were conducted on R 4.4.1, GraphPad Prism 10 and Adobe Illustrator 2024. Statistical comparisons among multiple groups were performed using one-way ANOVA followed by Tukey’s *post hoc* test, with significance defined as *P* <0 .05. Time-course and repeated-measures data were analyzed using linear mixed-effects models to assess treatment effects and interactions over time. Differentially expressed genes in transcriptomic analyses were identified using DESeq2 with a log2 fold change >2 and a false discovery rate < 0.05, and pathway enrichment analyses were performed using standard enrichment metrics.

## Results

### *Bacillus* and *Trichoderma* positively correlate in soil

To investigate *Trichoderma* and *Bacillus* distribution, we analyzed 1680 soil metagenomes from various environments (Fig. 1A and B), including agricultural soil (48.9%), rhizosphere (30%), wetland (4.1%), grassland (2%), and unknown soil (15%). Correlation analysis revealed significant positive correlation overall (*r* = 0.19, *P* < .001, n = 1680, Fig. 1C). Both *Bacillus* and *Trichoderma* showed negative or nonsignificant correlations with other agriculturally important microorganisms, including *Aspergillus, Glomus, Pseudomonas, Streptomyces, Actinomyces*, and *Enterobacter* (Fig S3), highlighting their strong positive correlation compared with other analyzed genera. Further analysis of individual soil types revealed significant positive correlations across all environments, with grassland showing the strongest significance (*r* = 0.48, *P* = .004) despite having the smallest sample size (n = 34), followed by agricultural soil (*r* = 0.25, *P* < .001, n = 821), and rhizosphere (*r* = 0.17, *P* < .001, n = 504).

This widespread positive correlation suggested that members of the *Bacillus* and *Trichoderma* genera may have developed a mechanism for mutual adaptation and coexistence through certain interactions, rather than competition.

### Co-inoculation of *B. velezensis* and *T. guizhouens*e enhance disease suppression and plant growth promotion

**Figure 2:**
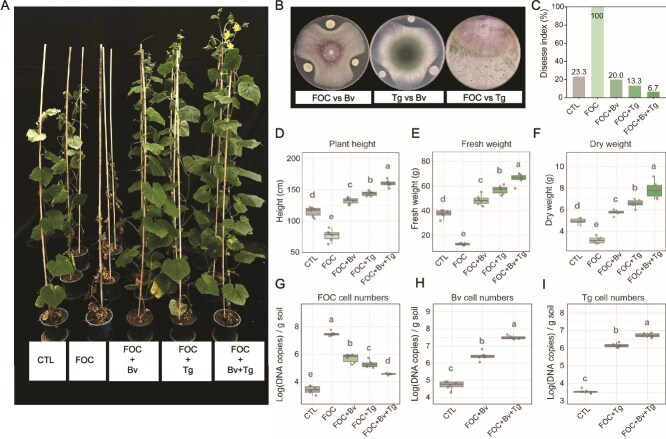
Co-inoculation of *T. guizhouense* and *B. velezensis* enhances disease suppression and cucumber growth. (**A**) Cucumber plants with different treatments. CTL: control, no inoculation. (**B**) The interactions between *T. guizhouense* (Tg), *B. velezensis* (Bv) and *Fusarium oxysporum f.* sp. *cucumerinum* (FOC). The diameter of petri dish plate: 9 cm. (**C**) Disease index based on foliar symptoms (leaf growing and wilting), where lower values indicate reduced disease severity. (D–F) Plant growth parameters: plant height (**D**), fresh weight (**E**) and dry weight (**F**) of cucumber plants. (**G–I**) Microbial cell numbers in soil quantified by RT-qPCR: FOC (**G**), Bv (**H**), and Tg (**I**). Bars represent ± s.d. (n = 6). Significance test was performed using one-way ANOVA followed by Tukey’s *post hoc* test. Different letters indicate statistically significant (*P* < .05) differences.

The antagonistic interactions among *B. velezensis, T. guizhouense*, and FOC were first observed on agar medium (Fig. 2B). *B. velezensis* inhibited both fungi, as expected based on the production of various secondary metabolites [40], whereas *T. guizhouense* suppressed FOC growth through mycoparasitism via secretion of chitinase and protease [41]. These agar-based interactions, though simplified, established based antagonistic interactions which contrast with the enhanced growth in subsequent plant experiments. Given the well-studied capabilities of *B. velezensis* and *T. guizhouense* as PGPM individually [17, 42, 43], we further investigated the effects of their co-inoculation on plant growth in pot experiments.

We investigated effects of co-inoculation on cucumber growth in pot experiments (Fig. 2A). After 8-week growth, FOC-inoculated plants showed severe yellowing of leaves and growth inhibition, with disease index of 100% (Fig. 2C). Individual inoculation with either *B. velezensis* or *T. guizhouense* alleviated FOC-induced damage, improving plant height, fresh weight, and dry weight (Figs 2D–F). Despite the antagonism on agar medium, co-inoculation (FOC + Bv + Tg) achieved the lowest disease index (6.7%) and best plant performance across all treatments. Microbial populations aligned with plant growth: FOC numbers were lowest in FOC + Bv + Tg treatment (Fig. 2G), whereas Bv and Tg numbers maintained relatively high abundance (Fig. 2H and I), suggesting these beneficial microorganisms overcome their direct competitive interactions when confronting a shared pathogen, maintaining stable co-occurrence and coordinated pathogen suppression.

### FOC acts as the primary target in the three-species interaction

**Figure 3:**
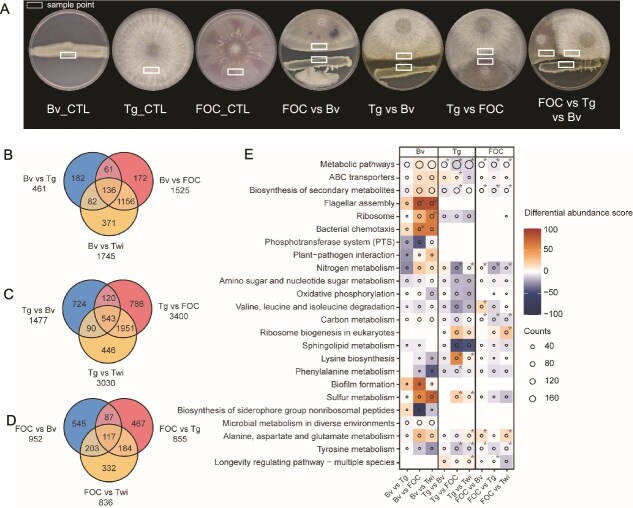
Transcriptome analysis of *B. velezensis, T. guizhouense* and FOC during microbial interactions. (**A**) Schematic representation of sampling positions for transcriptome analysis from interaction zones. Rectangle boxes indicated sampling areas. The diameter of petri dish plate: 9 cm. (B–D) Venn diagrams among overlap of differentially expressed genes (DEGs) *B. velezensis* (**B**), *T. guizhouense* (**C**) and FOC (**D**) across interactions (Twi = Bv vs Tg vs FOC). DEGs were identified using DESeq2 with LFC >2, FDR < 0.05. Numbers in each section represent gene counts that were either unique or shared to specific interactions. (**E**) KEGG pathway enrichment analysis showing differentially regulated pathways across all treatments. Circle size indicates the number of DEGs (counts) mapped to each pathway. Color scale represents differential abundance score: red indicates pathway upregulation, blue indicates downregulation, with intensity proportional to the magnitude of enrichment. Asterisks denote statistical significance: ^*^P < .05.

Following enhanced disease suppression through co-inoculation, we performed transcriptome analysis to dissect the molecular interactions. We sampled interaction zones from pairwise combinations on agar medium (Fig. 3A) to examine transcriptional responses, though acknowledging this simplified setup cannot replicate soil complexity, it allows controlled examination of direct pairwise interactions.

Differential gene expression analysis showed that both PGPMs exhibited strong transcriptional responses against FOC. *B. velezensis* exhibited extensive transcriptional changes when interacting with FOC (1525 genes) or in the presence of both fungi (1745 genes), with 1156 genes commonly regulated in both treatments (Fig. 3B). Similarly, *T. guizhouense* displayed substantial responses when challenged with FOC (3400 genes) or both microorganisms (3030 genes), sharing 1951 regulated genes (Fig. 3C). The direct interaction between *B. velezensis* and *T. guizhouense* revealed relatively fewer changes (461 and 1477 genes, respectively), supporting their potential coexistence despite agar-based antagonism. FOC showed consistent response to *B. velezensis* (952 genes), *T. guizhouense* (855 genes), or both (836 genes), with distinct gene sets responding to each beneficial microorganism (Fig. 3D).

Principal coordinate analysis (PCoA) further confirmed both *B. velezensis* and *T. guizhouense* primarily responded to FOC in the tri-species interactions (Figs S4A and B, D, and E). In contrast, FOC exhibited distinct responses to different treatments (Fig S4C and F). Whereas Bv and Tg primarily responded to pathogen presence, Bv showed the greatest magnitude of transcriptional changes (Fig. 3E), likely due to exposure to FOC-secreted metabolites such as fusaric acid, suggesting complex reciprocal interactions rather than a simple antagonistic targeting.

### σ^B^ mediates stress response in *B. velezensis* during interaction with fungi

To elucidate molecular mechanisms underlying transcriptional responses, we performed pathway enrichment analysis (Fig. 3E). *B. velezensis* demonstrated upregulation of flagellar assembly and bacterial chemotaxis, particularly when confronting FOC, suggesting enhanced movement ability to detect and respond to environmental signals [44, 45]. Additionally, *B. velezensis* showed upregulation of biofilm formation and sulfur metabolism pathways, indicating competitive strategies through spatial occupation and sulfur-containing metabolites production [46, 47]. *T. guizhouense* exhibited downregulated sphingolipid metabolism and upregulated lysine biosynthesis, indicating membrane restructuring and enhanced amino acid metabolism [48]. Both *Bacillus* and *Trichoderma* exhibited substantial modulation of secondary metabolite biosynthesis pathways [49]. Basic metabolic processes including carbon metabolism and amino acid metabolism were regulated across all three species, with ribosome-related pathways active in both *B. velezensis* and *T. guizhouense*. Altered ABC transporters across all species suggest potential metabolite exchange [50]. Whereas *B. velezensis* and *T. guizhouense* showed specific pathway regulations, FOC exhibited relatively consistent responses.

**Figure 4:**
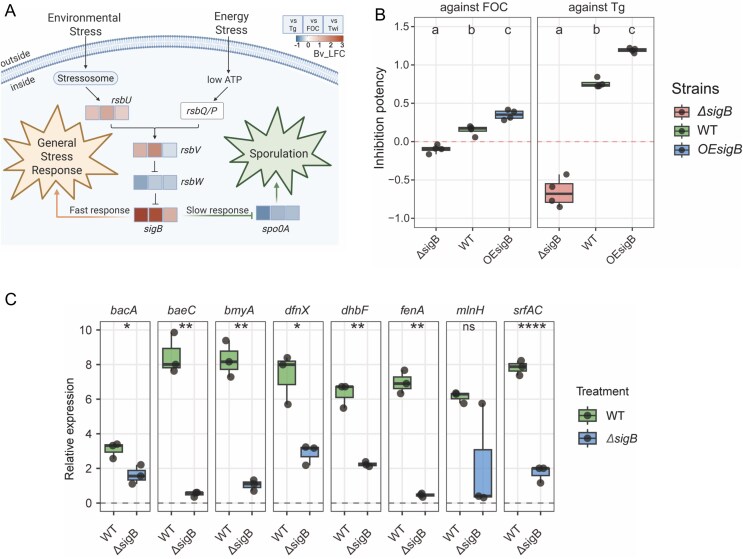
SigB regulated secondary metabolite production to mediate the rapid response of*. velezensis* to fungi. (**A**) The pathway of global regulator SigB, which mediates both fast and slow responses through regulatory networks. Heatmap shows gene expression changes (log_2_ fold change, LFC) of *B. velezensis* in response to the interactions with different treatment. (**B**) The inhibition potency of Δ*sigB*, WT and *OEsigB* strains against FOC and Tg. *OEsigB*: *sigB* gene overexpress stain. Bars represent ± s.d. (n = 4). Significance test was performed using one-way ANOVA followed by Tukey’s posthoc test. Different letters indicate statistically significant (*P* < .05). (**C**) Relative expression of biosynthetic genes for secondary metabolites in *B. velezensis* during interaction with fungi, quantified by RT-qPCR. Bars represent ± s.d. (n = 3). Statistical analysis was performed using *t* test. (^*^P < .05, ^*^^*^P < .01, ^*^^*^^*^^*^P < .0001, ns indicates no significant difference). The secondary metabolites of *B. velezensis*: *bacA*: Bacilysin; *baeC*: Bacillaene; *bmyA*: Bacillomycin; *dfnX*: Difficidin; *dhbF*: Bacillibactin; *fenA*: Fengycin; *mlnH*: Macrolactin; *srfAC*: Surfactin.

Among the differentially expressed genes, we identified significant upregulation of *sigB* transcription that encodes the sigma factor σ^B^ (Sigma B) in *B. velezensis*. As σ^B^ functions as a global regulator coordinating stress responses in Gram-positive bacteria [51], we investigated its potential role in mediating *B. velezensis* adaptation in the tri-species interaction environment (Fig. 4A). Environmental stress signals are perceived through the stressosome, activating RsbU, which subsequently regulates RsbV and RsbW, ultimately controlling SigB activity [52]. When *B. velezensis* confronted *T. guizhouense* or FOC, *sigB* gene showed significant transcriptional upregulation, potentially triggering rapid stress response, whereas *spo0A* gene transcription downregulation indicated prioritization of rapid response over sporulation [53]. To validate the role of SigB, we constructed Δ*sigB* mutant and *OEsigB* overexpression strains. During interactions with both fungi, Δ*sigB* showed diminished antifungal metabolite production in inhibition assays, whereas *OEsigB* exhibited enhanced antagonistic activity compared with WT (Fig. 4B and Fig S5). This was further supported by examining the expression of secondary metabolite biosynthetic genes involved *B. velezensis* (Fig. 4C). Collectively, these results demonstrate that SigB is a key regulator mediating rapid stress response in *B. velezensis* during fungal interactions.

### Surfactin enhances T22azaphilone production in *T. guizhouense*

**Figure 5:**
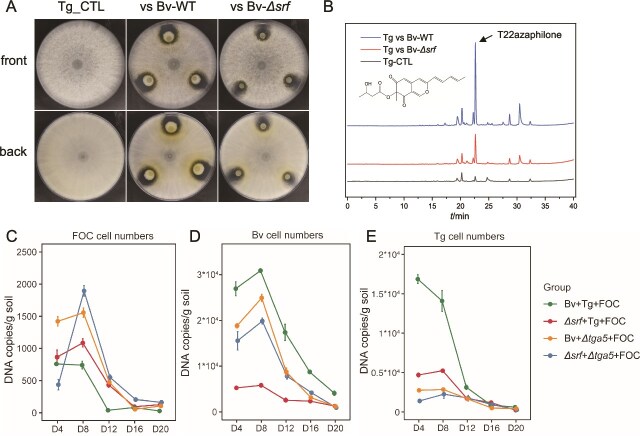
Surfactin induced the protection mechanism of *T. guizhouense*. (**A**) The interactions between *T. guizhouense* and *B. velezensis* wild type (WT) or surfactin-deficient mutant *(*Δ*srf)* strains. The diameter of petri dish plate is 9 cm. (**B**) T22azaphilone production in *T. guizhouense* and quantified by HPLC (peak time = 22.5 min). (**C–E**) Microbial population dynamics in sterilized soil over 20 days, showing FOC (**C**), *B. velezensis* (**D**), and *T. guizhouense* (**E**) cell numbers quantified by qPCR across different mutant combinations. Treatment groups: Bv + Tg + FOC (all wild-type), Δ*srf* + Tg + FOC (*B. velezensis* surfactin mutant), Bv + Δtga5 + FOC (*T. guizhouense* T22azaphilone mutant), and Δ*srf* + Δ*tga5* + FOC (combination of mutant strains). Time points indicate days post-inoculation (D4 = day 4, D8 = day 8, D12 = day 12, D16 = day 16, D20 = day 20). Lines represent mean values ± s.d. (n = 6). Statistical analysis using linear mixed-effects models (LME) revealed significant treatment effects and treatment-time interactions (all *P* < .001).

Given that σ^B^-mediated stress response activates biosynthetic gene clusters, we tested various *B. velezensis* secondary metabolite mutants (Fig S6). During interaction with *T. guizhouense, B. velezensis* induced the production of T22azaphilone, a yellow compound protecting *T. guizhouense* against oxidative stress and fungicides [26]. In contrast, T22azaphilone production was less pronounced with surfactin-deficient Δ*srf* strain (Fig. 5A), as quantified by HPLC (Fig. 5B). Surfactin plays vital roles in collective motility (swarming and sliding) [54–56], biofilm formation [57, 58], microbial interactions [59–61], and intraspecies communications [62, 63] in *Bacillus*. RT-qPCR analysis revealed that *tga5* expression, involved in T22azaphilone production, was significantly reduced in *T. guizhouense* in the presence of Δ*srf* compared with the wild-type *B. velezensis* (Fig S7). Further experiments demonstrated that *T. guizhouense* produces substantial peroxide (brown) and superoxide (blue) during interactions with *B. velezensis* and FOC (Fig S8).

To validate that surfactin induces T22azaphilone-mediated production in *T. guizhouense*, we co-inoculated different combinations including the Δ*srf* and Δ*tga5* mutants in bare sterilized soil (without plants) over 20 days. Linear mixed-effects model analysis revealed significant treatment effects for all three microorganisms (LME, Type III ANOVA, all *P* < .001) with significant treatment-time interactions. *B. velezensis* maintained the highest abundance due to its rapid growth (Fig. 5D), with wild-type combinations significantly outperforming mutant combinations (Estimate = 5936.35, t (95) = 5.59, *P* < .001 at D8). *T. guizhouense* populations in Bv + Tg + FOC were significantly higher than other combinations (Estimate = 11244.46, t (95) = 21.04, *P* < .001 at D8, Fig. 5E), supporting the hypothesis that *T. guizhouense* WT maintained higher populations in the presence of *B. velezensis* WT due to surfactin-mediated T22azaphilone induction. In contrast, *T. guizhouense* levels were reduced when interacting with Δ*srf*, and displayed the lowest abundance when the Δ*tga5* strain lacking T22azaphilone production (Fig. 5E). Correspondingly, FOC populations were lowest in Bv + Tg + FOC treatment with progressive increases when surfactin or T22azaphilone was inactivated (Estimate = −1153.39, t (95) = −17.17, *P* < .001 at D8, Fig. 5C).

In conclusion, these experiments revealed that bacterial and fungal secondary metabolites strengthening abundance of PGPM in soil, potentially enhancing their collective antagonism against the pathogen.

### Fusaric acid-mediated inhibition of *B. velezensis* is prevented by *T. guizhouense*

In addition to the PGPM-mediated pathogen suppression, we examined pathogen-derived factors affecting microbial interactions. Fusaric acid (FA), a mycotoxin with low to moderate toxicity against plants and various microorganisms, is a key virulence factor produced by *Fusarium* sp. that plays critical roles in plant pathogenesis [64, 65]. To explore whether FOC inhibits either PGPMs, we investigated FA influence using standard FA, which was validated to be identical to FA in FOC supernatant by HPLC (Fig S2).

**Figure 6:**
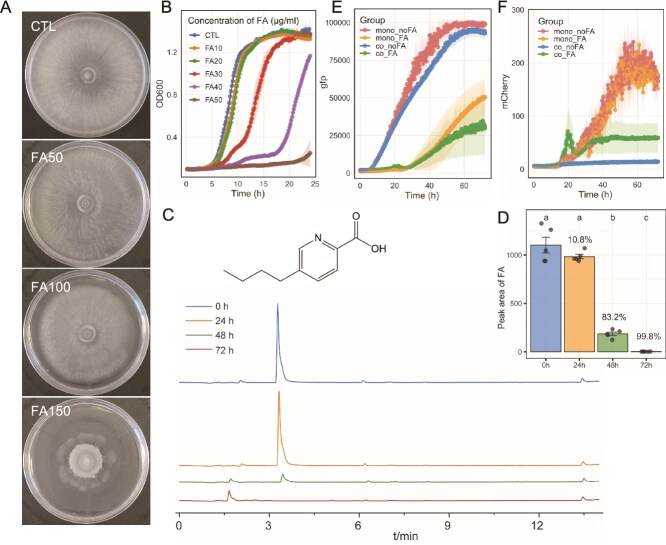
Fusaric acid maintains stability in the tri-species microbial system. (**A**) The growth of *T. guizhouense* in different concentration of fusaric acid. The concentration of fusaric acid: 0, 50, 100, 150 μg mL^−1^. The diameter of petri dish plate is 9 cm. (**B**) The growth curve of *B. velezensis* in LB medium supplemented with fusaric acid (0, 10, 20, 30, 40, 50 μg mL^−1^). Bars represent ± s.d. (n = 3). (**C**) Degradation of fusaric acid by *T. guizhouense* in minimal medium over 72 h, quantified by LCMS (peak time = 3.1 min). (**D**) Quantification of fusaric acid remaining at each time point based on HPLC-MS peak area integration. Percentages indicate the proportion of fusaric acid degraded by *T. guizhouense* at each time point. Bars represent ± s.d. (n = 4). Significance test was performed using one-way ANOVA followed by Tukey’s posthoc test. Different letters indicate statistically significant (*P* < .05) differences. (**E–F**) Growth dynamics of *B. velezensis* (**E**, monitored by GFP fluorescence) and *T. guizhouense* (**F**, monitored by mCherry fluorescence) in monoculture and coculture conditions with or without fusaric acid (30 μg mL^−1^). Treatments: Mono_noFA, monoculture without fusaric acid. Mono_FA: monoculture with fusaric acid 30 μg mL^−1^. Co_noFA: *B. velezensis* and *T. guizhouense* coculture without fusaric acid. Co_FA: *B. velezensis* and *T*.* guizhouense* coculture with fusaric acid 30 μg mL^−1^. Bars represent ± s.d. (n = 6).

Tolerance assays revealed striking differences in FA resistanc*e. T. guizhouense* showed robust tolerance to FA concentrations up to 100 μg mL^−1^ without growth inhibition (Fig. 6A). In contrast, *B. velezensis* showed growth delays at FA concentrations exceeding 20 μg mL^−1^, although it maintained growth capacity below 50 μg mL^−1^ of FA in the first 24 h (Fig. 6B, S9A). Previous studies reported potential of *Trichoderma* to degrade FA [66]. To validated this, we co-cultured *T. guizhouense* with 100 μg mL^−1^ FA and quantified FA concentration every 24 h using LC–MS (Fig. 6C). *T. guizhouense* exhibited efficient FA degradation capacity (Fig. 6D); only 10.8% degraded during the first 24 h spore germination, but degradation accelerated dramatically to 83.2% by 48 h, with nearly complete degradation (99.8%) after 72 h. This suggests that *T. guizhouense* exerts dual benefits by suppressing FOC whereas eliminating phytotoxic FA.

To further examine the interactions between the two PGPMs in the presence of FA, we used *B. velezensis* and *T. guizhouense* constitutively expressing GFP and mCherry proteins, respectively. In coculture without FA, *T. guizhouense* was unable to growth, whereas *B. velezensis* growth was unaffected (Figs 6E–F and S9, co_noFA). We attributed this to their different growth rates, as *B. velezensis* reached stable phase within 24 h, severely inhibiting *T. guizhouense* spore germination. However, with 30 μg mL ^−1^ FA supplementation, we observed stable coexistence (Figs 6E–F and Fig. S9, co_FA). We hypothesized that FA delayed *B. velezensis* growth (Fig. 6B and E), whereas FA-tolerant *T. guizhouense* spores had sufficient time and nutrients to germinate (Fig. 6F). Between 24 and 48 h, *T. guizhouense* began degrading FA, gradually alleviating *B. velezensis* inhibition and allowing normal growth resumption.

In conclusion, FA facilitates stable coexistence at certain FA concentrations. *T. guizhouense* degradation of FA enables collective growth of both PGPMs for effective pathogen suppression and plant growth promotion.

## Discussion

**Figure 7:**
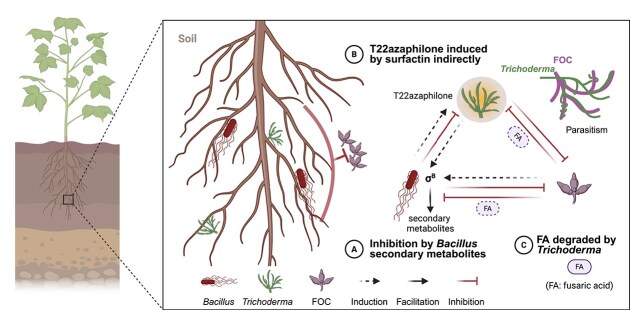
Summary diagram: secondary metabolites mediated molecular interactions and microbial dynamics. (**A**) *B. velezensis* secondary metabolites inhibit the growth of *T. guizhouense* and FOC. Surfactin induces T22azaphilone (yellow compound) production, which protects *T. guizhouense* from oxidative stress. (**B**) *T. guizhouense* suppresses FOC through mycoparasitism by degrading fungal hyphae. (**C**) Fusaric acid temporarily inhibits *B. velezensis* growth, allowing *T. guizhouense* to establish first, after which *T. guizhouense* degrades fusaric acid. Plants benefit from reduced pathogen pressure and enhanced growth-promoting activities from beneficial microorganisms, ultimately improving plant growth and health.

In this study, we found that *B. velezensis* and *T. guizhouense* jointly suppress FOC in soil, ultimately leading to suppression of pathogenesis and enhanced plant growth (Fig. 7). *B. velezensis* initiates rapid σ^B^-mediated response by upregulating secondary metabolite production. One of these secondary metabolites, surfactin induces T22azaphilone production in *T. guizhouense*, which provides self-protection and damage reduction for the fungus. In parallel, fusaric acid secreted by FOC delays *B. velezensis* growth which *T. guizhouense* reverses by degrading fusaric acid. Collectively, *T. guizhouense* inhibits FOC through mycoparasitism and hyphal degradation, whereas *B. velezensis* suppresses the pathogen via secondary metabolite production, together establishing a balanced PGPMs community. Our study reveals the molecular details of beneficial bacterial-fungi interactions, providing insights for designing microbial consortia.

Our global soil metagenome analysis revealed significant positive correlations between *Bacillus* and *Trichoderma* genera across various soil types, which showcases for beneficial microorganisms naturally co-occur in diverse environments. This suggests that these microorganisms may have evolved mechanisms to reduce direct competitive pressure. The genus-level pattern implies potential species-wide associations, though strain-level variation in secondary metabolite production may influence specific interaction outcomes. Even with a limited sample size, the strong positive correlation observed in grassland environments points that ecosystems high in organic matter foster complex and stable microbial networks [67]. The observed correlation hints at potential niche differentiation through spatial resource partitioning [68]. *Bacillus*, characterized by motility and chemotaxis, tend to colonize small pore spaces and root surfaces through biofilm formation, producing diffusible antimicrobial compounds [11, 69]. Whereas *Trichoderma*, with its hyphal network and mycoparasitic capabilities, may preferentially colonize organic matter and root surfaces. Such functional complementarity through resource partitioning could reduce direct competition and maintain synergistic biocontrol. However, direct spatial analysis is needed to confirm whether similar partitioning occurs in this interaction.

Although *T. guizhouense* and *B. velezensis* exhibit antagonism in vitro, their transition from antagonism to synergy in pot experiments offers a more realistic scenario of environmental interactions. Transcriptomic analysis revealed the molecular basis: σ^B^ global regulator mediated *B. velezensis* rapid response through upregulating secondary metabolites and chemotaxis, whereas maintain defensive measures like biofilm formation. Concurrently, *T. guizhouense* specifically induces T22azaphilone in response to surfactin, demonstrating cross-kingdom chemical signaling. These secondary metabolites function beyond simple antagonism, serving as chemical communication signals that mediate ecological relationships. Surfactin, plays multifunctional roles from antimicrobial activity to signaling [59–61]. T22azaphilone’s has dual protective role against both oxidative stress and fungicides [26], representing a unique adaptation mechanism compared to other known fungal defense strategies. The induction of protective mechanisms in *T. guizhouense* by surfactin demonstrates how *Bacillus* metabolites trigger adaptive responses in *Trichoderma*. Such cross-kingdom communication resembles previous observations where iturin A of *B. subtilis* C2 induces fungal (*Gliocladium roseum* and *T. harzianum*) chlamydospore formation at low concentration but suppressing growth at high concentration [70], supporting concentration-dependent effects that maintain microbial community homeostasis whereas retaining competitive capabilities.

Fusaric acid represents another example of a microbial derived metabolite with dual roles: beyond toxicity against the host, it influences community stability between *Bacillus* and *Trichoderma*. Delayed *B. velezensis* growth allows *T. guizhouense* to germinate and subsequently degrade fusaric acid, exemplifying temporal niche differentiation, a key mechanism allowing species coexistence in natural ecosystems [71, 72]. Similarly, soil bacteria are capable of degrading the toxic oxalic acid produced by *Sclerotinia sclerotiorum* that reduce its pathogenicity [12, 73]. Our previous research demonstrated that when *T. guizhouense* is cultured first, *B. velezensis* forms stable biofilms on *Trichoderma* hyphae [14], suggesting a possible mechanism underlying their coexistence and FOC suppression.

The temporal dynamics during the interaction of these three species suggest distinct phases (Fig. 7). The early stage is characterized by intense competition and stress responses, evidenced by σ^B^ activation and ROS production. As the community matures, the interaction transition towards a more stable state with fusaric acid degradation and biofilm formation. Such dynamic stabilization demonstrates how initial antagonistic relationships can transition into more balanced associations that maximize collective resilience. The transition from antagonism to cooperation challenges simplified views of microbial interactions as fixed and binary, suggesting that ecological context and temporal dynamics are equally important as species identity in determining functional outcomes in microbial consortia applications.

Despite identifying key molecular mechanisms underlying *Bacillus*-*Trichoderma* cooperation, several aspects warrant further investigation. The single-strain approach enabled detailed mechanistic dissection, though strain-level metabolic variation requires examination of examining multiple isolates to assess whether interactions represent species-wide or strain-specific phenomena. Our agar medium-based assays using PDA medium provided controlled laboratory conditions for identifying core interaction mechanisms, yet natural soil environments present additional complexity through heterogeneous nutrient distributions, diverse indigenous microbial communities, and spatial structures that may influence interaction outcomes. Additionally, both species produce numerous metabolites beyond surfactin and T22azaphilone examined here, which may also contribute to their ecological interactions. Future research should emphasize multistrain validation, soil microcosm experiments with natural microbial communities, and mechanistic dissection of chemical signaling under varying environmental conditions to advance practical applications of these consortia.

In conclusion, our findings revealed a sophisticated chemical dialogue affecting microbial community assembly in the rhizosphere and provides a foundation for designing effective microbial consortia for sustainable agriculture. The transition from antagonism to cooperation through σ^B^-mediated metabolic reprograming, surfactin-induced protective responses, and temporal niche differentiation. This demonstrates how ecological communities self-organize and achieve balance under different environmental conditions, with important implications for understanding soil ecosystem assembly. For biocontrol applications, successful microbial combinations require carefully timed species introduction and consideration of metabolic interactions, rather than simply combining organisms with desired traits.

## Supplementary Material

Supplementary_materials_wraf283

Data_S1_Metagenomic_samples_list_wraf283

## Data Availability

Supporting data for all results presented in this paper are contained within the manuscript and supplementary materials. Transcriptome sequencing datasets have been submitted to the NCBI Sequence Read Archive (SRA) database under BioProject PRJNA1201065. Soil metagenomic sequence samples list can be found in Supplementary Data S1. All other data generated and analyzed during this study can be requested form the corresponding author.
