# Supplementary material for: Metabolite interactions mediate beneficial alliances between Bacillus and Trichoderma for effective Fusarium wilt control
Source: ISME J. 2025 Dec 27;20(1):wraf283. doi: 10.1093/ismejo/wraf283 (PMC12887306; doi:10.1093/ismejo/wraf283)
Supplement: Supplementary_materials_wraf283 [file supplementary_materials_wraf283.pdf]

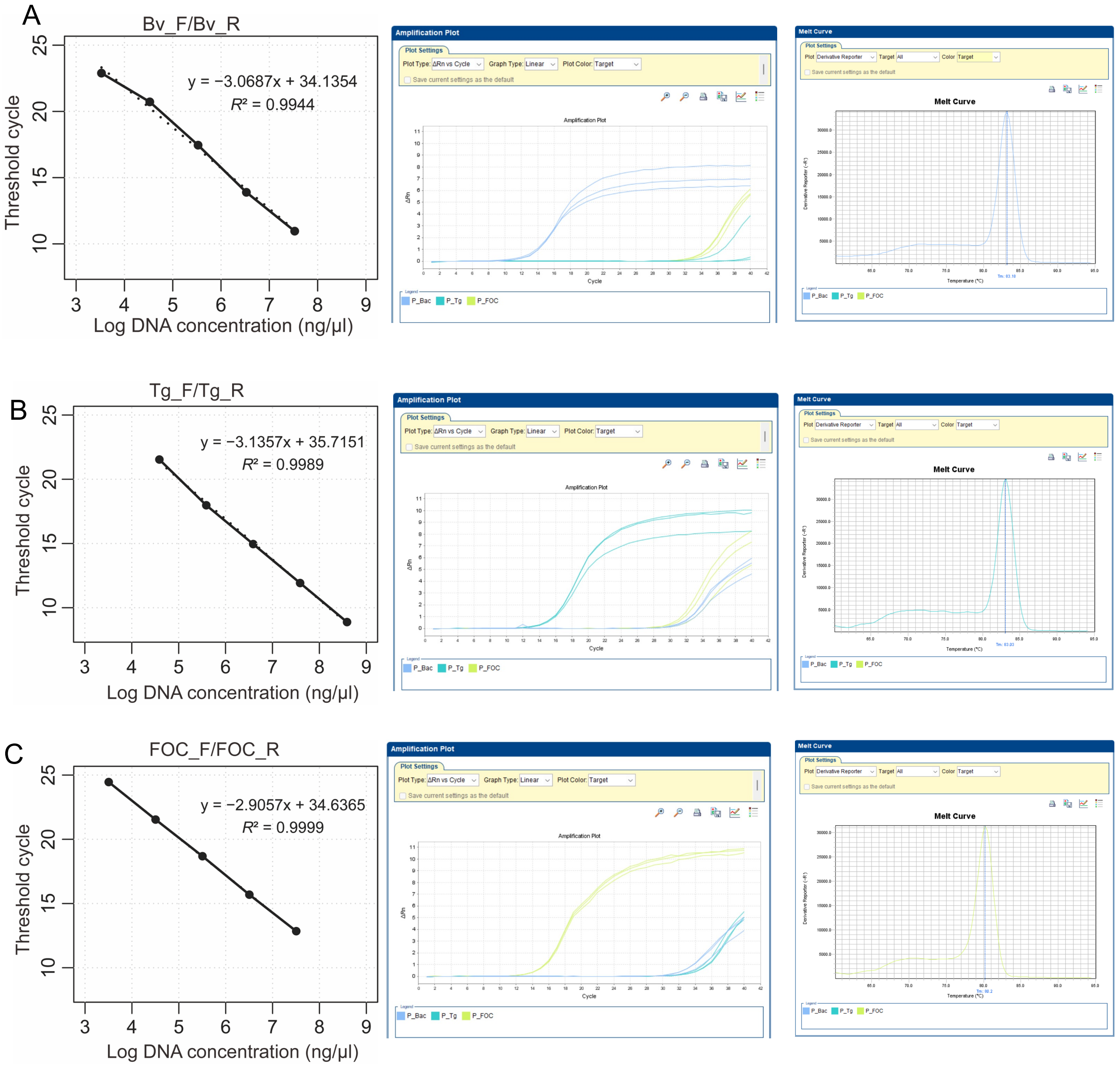

Fig. S1 PCR standard curves, amplification plots, and melting curves for quantifying *B. velezensis* (A), *T. guizhouense* (B), and FOC (C) cell numbers in soil. Left panels: standard curves generated from serial dilutions of cloned target genes, showing linear relationship between Ct values and DNA concentration with high coefficients of determination (R^2 > 0.99). Middle panels: representative amplification plots. Right panels: melting curve analysis showing single peaks, confirming primer specificity.

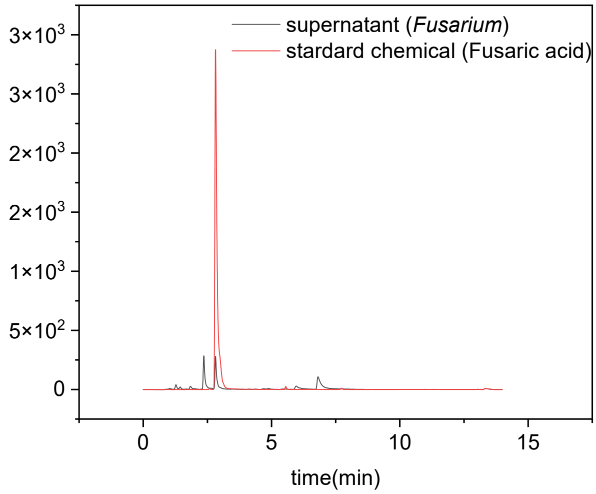

Fig. S2 Comparison between standard fusaric acid and supernatant by HPLC-MS (peak time: 3.1 min).

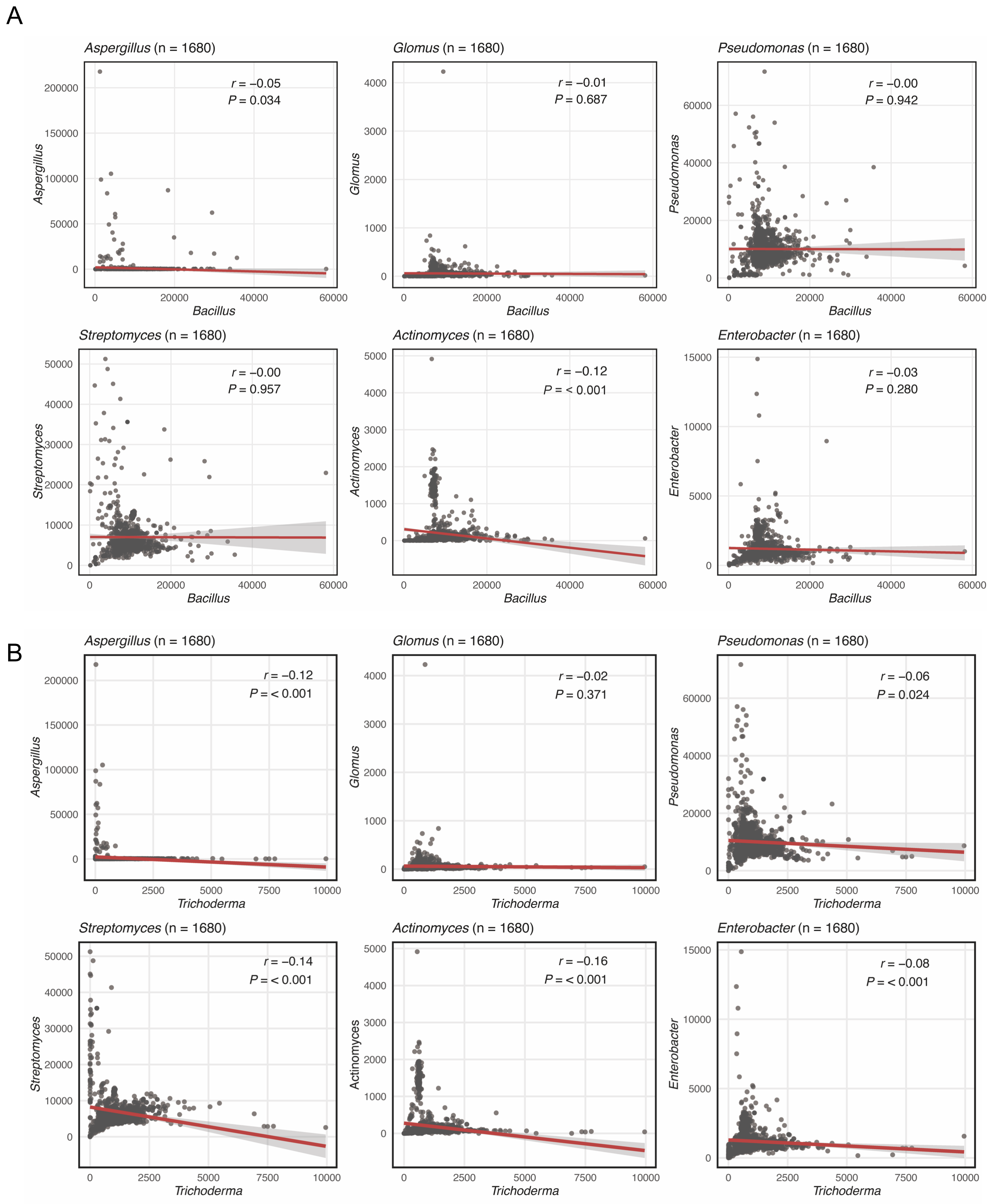

Fig. S3 Correlation analysis of *Bacillus* (A) and *Trichoderma* (B*)* with other agriculturally important microorganisms in global soil metagenomes. Pearson correlation analysis was performed to assess the correlation between the abundance of *Bacillus* or *Trichoderma* and other microorganisms (*Aspergillus*, *Glomus, Pseudomonas*, *Streptomyces*, *Actinomyces* and *Enterobacter*) across 1680 soil metagenomic samples. Each point represents one soil sample. Red lines indicate linear regression trends with 95% confidence intervals (gray shading). Correlation coefficients (*r*) and *P* values are shown in each panel.

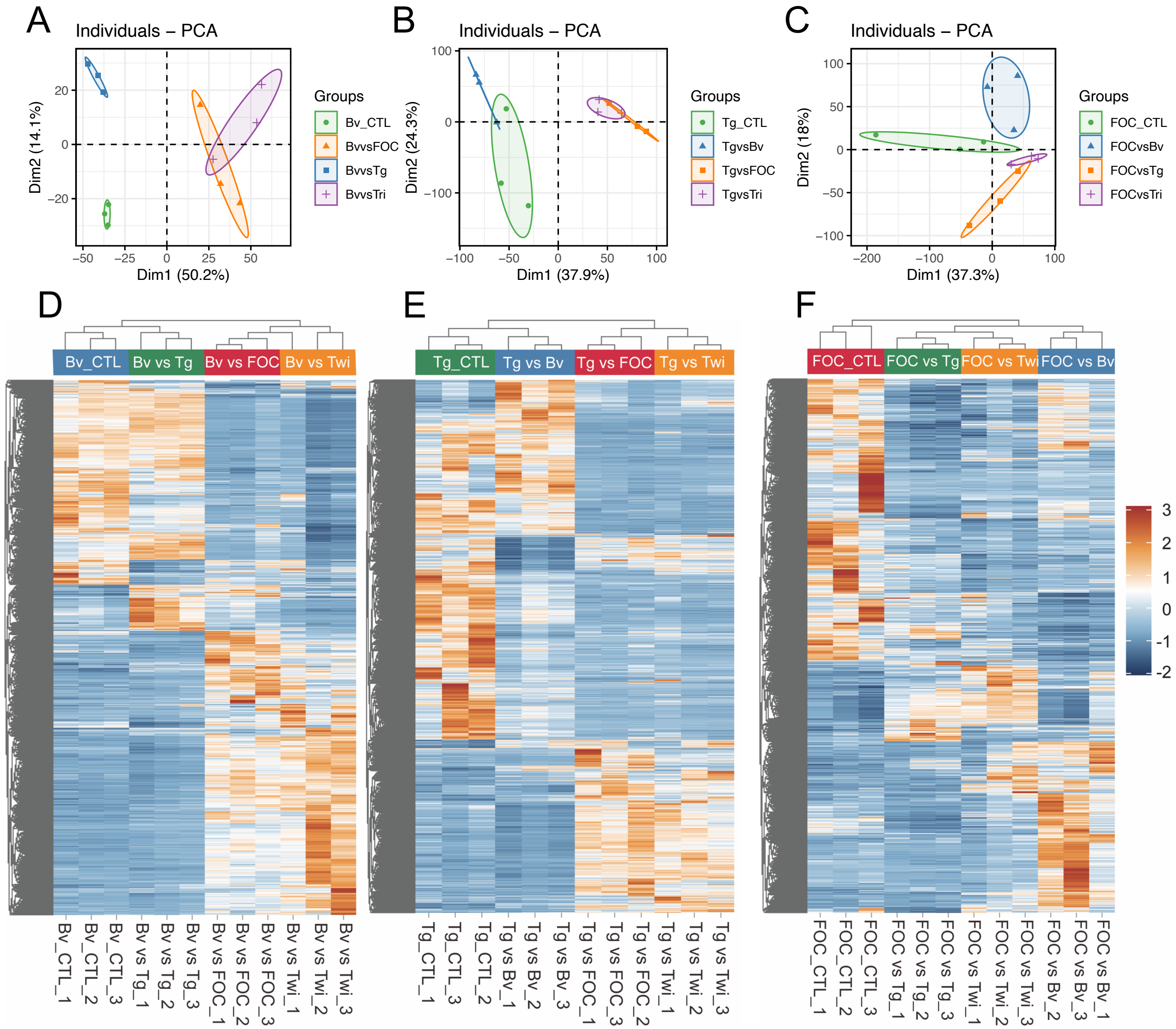

Fig. S4 Transcriptome analysis. (A-C) Principal Coordinate Analysis (PCoA) of gene expression profiles in *B. velezensis* (A), *T. guizhouense* (B), and FOC (C) based on Bray-Curtis dissimilarity matrices. Ellipses represent 95% confidence intervals for each treatment group. Percentages indicate the proportion of total variance explained by each principal coordinate axis. (D-F) The heatmaps showing differentially expressed genes (DEGs) in *B. velezensis* (D), *T. guizhouense* (E), and FOC (F) across different interaction conditions. Heatmaps display normalized expression values (blue = downregulated, red = upregulated) for genes meeting the criteria LFC > 2 and FDR < 0.05. Sample replicates (1-3) indicated at bottom.

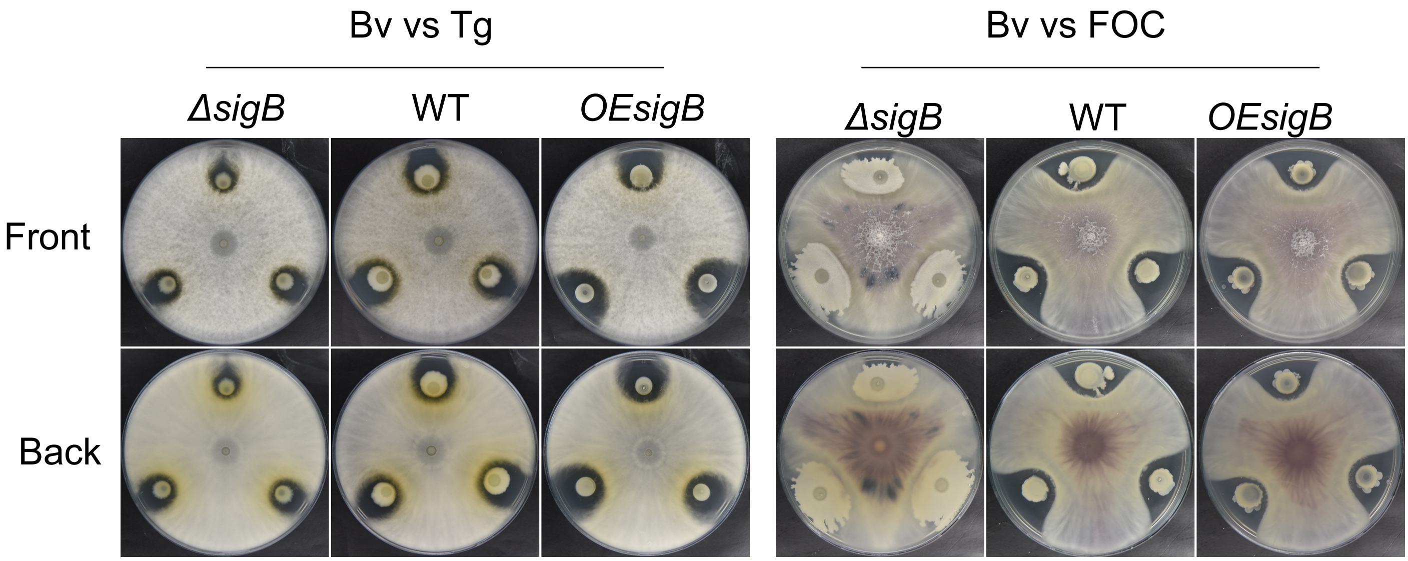

Fig. S5 The inhibition ability of *ΔsigB* and *OEsigB* to *T. guizhouense* and FOC. The diameter of Petri dish plate: 9 cm. OEsigB: *sigB* gene overexpress strain.

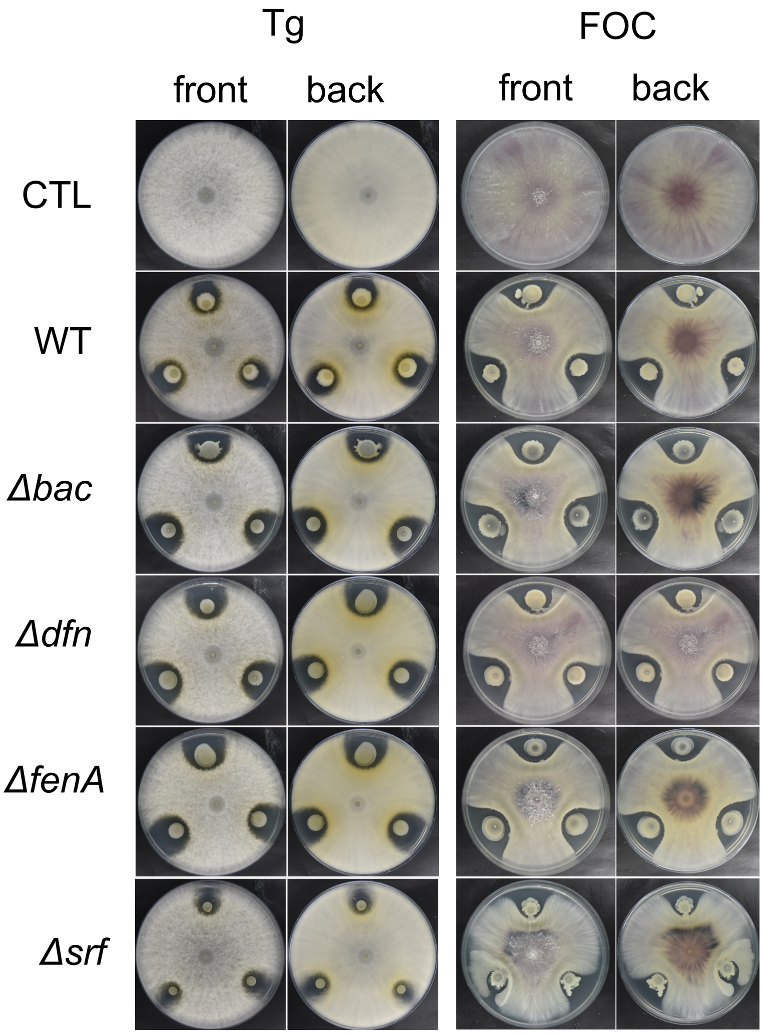

Fig. S6 The interaction between different *B. velezensis* mutants and two different fungi. The diameter of Petri dish plate: 9 cm. Gene: *bac,* bacillaene; *dfn*, difficidin; *fenA*, fengycin; *srf,* surfactin.

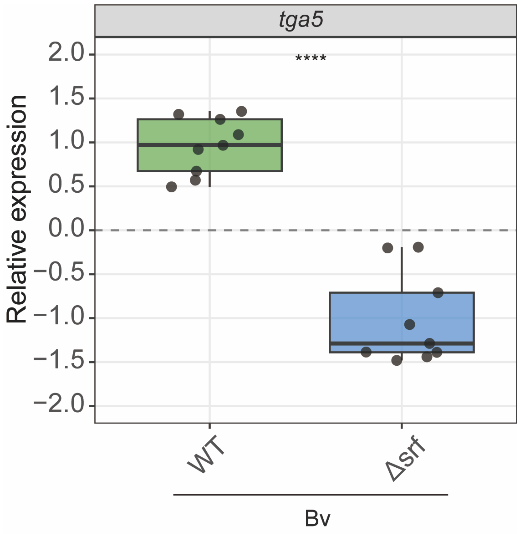

Fig. S7 The expression of T22azaphilone (*tga5*) in *T. guizhouense* during interacting with *B. velezensis* WT or *Δsrf*. Relative expression levels of *tga5* in *T. guizhouense* when co-cultured with *B. velezensis* wild-type (WT) or surfactin mutant (*Δsrf*) on plate confrontation assays, quantified by RT-qPCR. Bars represent ± s.d. (n=9). Statistical significance assessed by unpaired two-tailed Student's t-test: **** ($P \leq 0.0001$).

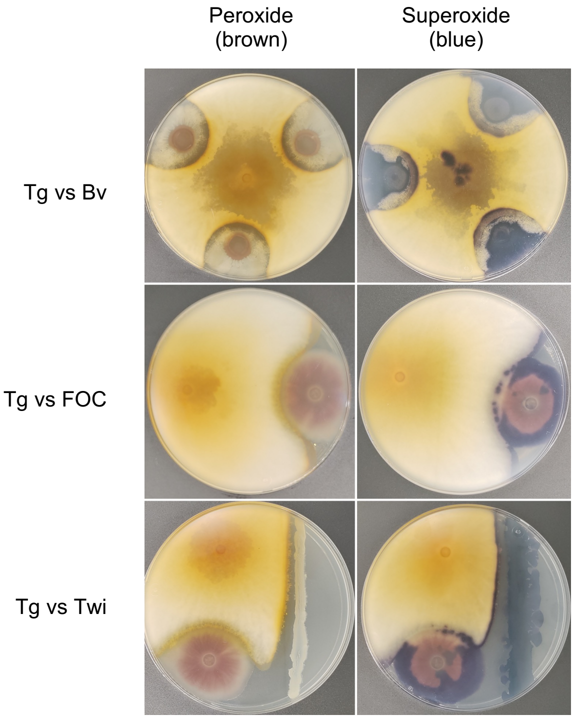

Fig. S8 Detection of peroxide and superoxide production in *T. guizhouense* during microbial interactions. Representative plates showing peroxide (H_2O_2, left panels) and superoxide (O_2^-, right panels) accumulation in *T. guizhouense* during interaction with *B. velezensis* (Tg vs Bv), FOC (Tg vs FOC), or both organisms simultaneously (Tg vs Twi = three-way interaction). ROS production was visualized using histochemical staining with 3,3'-diaminobenzidine (DAB) for peroxide (brown precipitate) and nitro blue tetrazolium (NBT) for superoxide (blue precipitate). Staining intensity correlates with ROS concentration, with darker coloration indicating higher oxidative stress. Petri dish diameter: 9 cm.

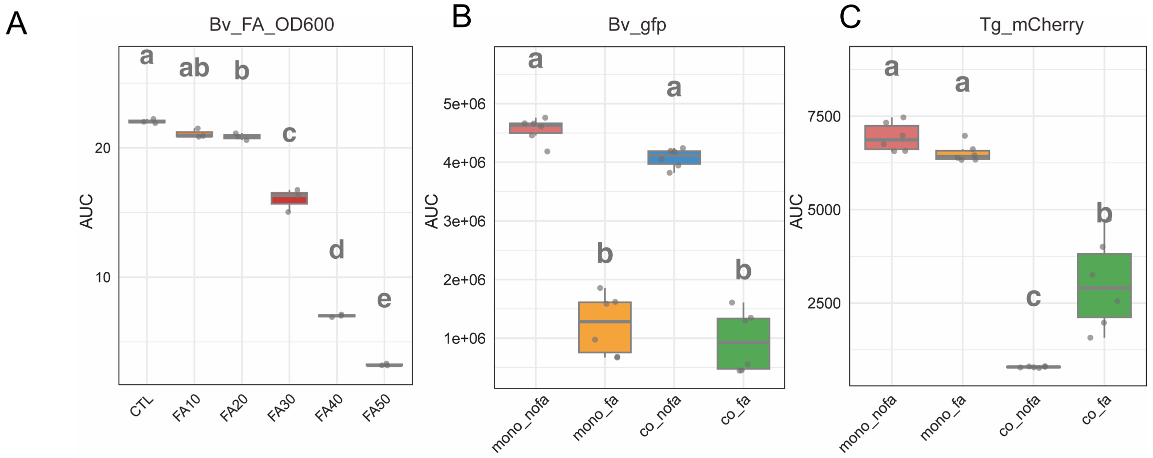

Fig. S9 Quantification of microbial growth using area under curve (AUC) analysis. (A) AUC values of *B. velezensis* growth curves (measured by OD_{600}) in response to different fusaric acid concentrations (0-50 μg/mL) over 72 hours. Bars represent ± s.d. (n=3). **(B-C)** AUC values quantifying growth of *B. velezensis* (B, monitored by GFP fluorescence) and *T. guizhouense* (C, monitored by mCherry fluorescence) in monoculture and coculture with or without fusaric acid (30 μg/mL). Treatment groups: mono_noFA (monoculture without fusaric acid), mono_fa (monoculture with fusaric acid), co_noFA (coculture without fusaric acid), co_fa (coculture with fusaric acid). Statistical significance assessed by one-way ANOVA followed by Tukey's post-hoc test. Different letters indicate significant differences ($P < 0.05$).

Table S1. Primers used in this study

Primers	Sequence (From 5' to 3')	Experimental purpose
**Strains construction**		
sigB_UF	CCGGTGGTTCGTGAAAAGC	To amplify the upstream region of *sigB* gene in *B. velzensis*
sigB_UR	CGTTACGTTATTAGTTATCGACGAGTGTTTCCTGGGC	To amplify the upstream region of *sigB* gene in *B. velzensis*
Spc_F	GCCCAGGAAACACTCGTCGATAACTAATAACGTAACG	Cloning
Spc_R	GTCCGCATCCGCTTCGATCGTATAATGTATGCTATA	Cloning
sigB_DF	TATAGCATACATTATACGATCGAAGCGGATGCGGAC	To amplify the downstream region of *sigB* gene in *B. velzensis*
sigB_DR	GACGGTATTGCCGTCTTCC	To amplify the downstream region of *sigB* gene in *B. velzensis*
sigB_F	GCAGCACGGGTAAAAAGCC	To confirm deletion of *sigB* gene in *B. velezensis*
sigB_R	CCATTGAGCCGGTCTGCT	To confirm deletion of *sigB* gene in *B. velezensis*

Classified as Internal | Intern

OEsigB_F	CGGAATTCATGGCACAACCATCAAAAACTAC (blue site: EcoR I)	To amplify *sigB* gene in *B.velezensis*
OEsigB_R	CGCGGATCCTTACATTAGCTCCATGGAGGGAT (blue site: BamH I)	To amplify *sigB* gene in *B.velezensis*

Relative gene expression quantification

RecA_F	AAAAAACAAAGTCGCTCCTCCG	To quantify the relative expression of housekeeping gene *recA* in *B. velezensis*
RecA_R	CGATATCCAGTTCAGTTCCAAG	To quantify the relative expression of housekeeping gene *recA* in *B. velezensis*
srfAC_F	CCGCAAACCTTTACTT	To quantify the relative expression of *srfAC* gene in *B. velezensis*
srfAC_R	AGGATGTCGGACCAGA	To quantify the relative expression of *srfAC* gene in *B. velezensis*
bmyA_F	AGTCTAAGTATTGGCGAAACGA	To quantify the relative expression of *bmyA* gene in *B. velezensis*
bmyA_R	ATTATGCTGAAAGTGAAGGGCG	To quantify the relative expression of *bmyA* gene in *B. velezensis*
fenA_F	AGCAAGGGAGACACGA	To quantify the relative expression of *fenA* gene in *B. velezensis*
fenA_R	CGAGAACCTGGGAGAC	To quantify the relative expression of *fenA* gene in *B. velezensis*
baeC_F	CGCACGGATTACATAC	To quantify the relative expression of *baeC* gene in *B. velezensis*
baeC_F	AACTCTTGTTTCGCTTC	To quantify the relative expression of *baeC* gene in *B. velezensis*
mlnH_F	GAAGGAAAGCGTAGTTG	To quantify the relative expression of *mlnH* gene in *B. velezensis*

Classified as Internal | Intern

mlnH_R	GAGGTTCGGAAGATGC	To quantify the relative expression of *mlnH* gene in *B. velezensis*
dfnX_F	AGCGGGAGATGAAGTG	To quantify the relative expression of *dfnX* gene in *B. velezensis*
dfnX_R	CCGACGGTTGTAATGC	To quantify the relative expression of *dfnX* gene in *B. velezensis*
bacA_F	CTGAAGGGACAAGCAGTGAG	To quantify the relative expression of *bacA* gene in *B. velezensis*
bacA_R	GATAGGAGACGGGTGGGATA	To quantify the relative expression of *bacA* gene in *B. velezensis*
dhbF_F	AGAGGTTTCGCTATTGG	To quantify the relative expression of *dhbF* gene in *B. velezensis*
dhbF_R	TTCGGCTTGTATGTTCC	To quantify the relative expression of *dhbF* gene in *B. velezensis*
rsbU_F	TGAACATCAGGTTCCTCCGG	To quantify the relative expression of *rsbU* gene in *B. velezensis*
rsbU_R	CCCGTAGCCAATCATGACT	To quantify the relative expression of *rsbU* gene in *B. velezensis*
rsbV_F	ATGTATACTCAGCTCCGGTG	To quantify the relative expression of *rsbV* gene in *B. velezensis*
rsbV_R	GCCTAAACCCGTACTGTCC	To quantify the relative expression of *rsbV* gene in *B. velezensis*
rsbW_F	TCCGGCCGAACCGGAATAT	To quantify the relative expression of *rsbW* gene in *B. velezensis*
rsbW_R	CGTTTGTACAAGCTTCGCT	To quantify the relative expression of *rsbW* gene in *B. velezensis*
sigB_F	AATGGCGGTGGATCAGCT	To quantify the relative expression of *sigB* gene in *B. velezensis*
sigB_R	CGACAGACAGCGCCTGATAG	To quantify the relative expression of *sigB* gene in *B. velezensis*
spo0A_F	GAGCTCTGATTCCCGCAGA	To quantify the relative expression of *spo0A* gene in *B. velezensis*

Classified as Internal | Intern

spo0A_R	GAGCTCTGATTCCCGCAGA	To quantify the relative expression of *spo0A* gene in *B. velezensis*
tef_F	TACAAGATCGGTGGTATTGGAACA	To quantify the relative expression of housekeeping gene *tef* in *T. guizhouense*
tef_R	AGCTGCTCGTGGTGCATCTC	To quantify the relative expression of housekeeping gene *tef* in *T. guizhouense*
tga5_F	GCTGGATGAGATGCGGAC	To quantify the relative expression of *tga5* gene in *T. guizhouense*
tga5_R	TGTAAGAGGCGGGATGGTG	To quantify the relative expression of *tga5* gene in *T. guizhouense*

Cell numbers quantification

Primers	Sequence (From 5’ to 3’)	Locus Tag	Gene name	Experimental purpose
Bv_F	ATCAGGCGTTTAACCGCAAG	V529_40680	hypothetical protein	To quantify the cell numbers of *B. velezensis*
Bv_R	GGCTTCTAGCCTGGCTTTGA			
Tg_F	CTCCACCGGCTGGTGAAAG	A0O28_0109270	hypothetical protein	To quantify the cell numbers of *T. guizhouense*
Tg_R	AAGACCCATCCCAACGCG			
FOC_F	GTGCGATCATCCCAATTGGCA	FOYG_13707	hypothetical protein	To quantify the cell numbers of FOC
FOC_R	CATCGCCGTCACCAAAACC			

Classified as Internal | Intern
